# A multicomponent ethanol response battery across a cumulative dose ethanol challenge reveals diminished adolescent rat ethanol responsivity relative to adults

**DOI:** 10.3389/adar.2023.11888

**Published:** 2023-12-21

**Authors:** Ryan P. Vetreno, Jeffrey Campbell, Fulton T. Crews

**Affiliations:** ^1^ Bowles Center for Alcohol Studies, School of Medicine, University of North Carolina at Chapel Hill, Chapel Hill, NC, United States; ^2^ Department of Psychiatry, School of Medicine, University of North Carolina at Chapel Hill, Chapel Hill, NC, United States; ^3^ Department of Pharmacology, School of Medicine, University of North Carolina at Chapel Hill, Chapel Hill, NC, United States

**Keywords:** acute alcohol, adolescence, alcohol sensitivity, development, hypothermia

## Abstract

Adolescence is a conserved developmental period associated with low alcohol responsivity, which can contribute to heavy drinking and development of an alcohol use disorder (AUD) later in life. To investigate ethanol responsivity between adolescent and adult rats, we developed an ethanol response battery (ERB) to assess acute ethanol responses across cumulative doses of ethanol during the rising phase of the blood ethanol curve. We tested the hypothesis that adolescent male and female rats would exhibit lower ethanol responsivity to a cumulative ethanol challenge relative to adults. Male and female adolescent (postnatal day [P]40) and adult (P85) Wistar rats underwent ERB assessment following consecutive doses of ethanol (i.e., 1.0, 1.0, and 1.0 g/kg) to produce cumulative ethanol doses of 0.0, 1.0, 2.0, and 3.0 g/kg. The ERB consisted of (1) the 6-point behavioral intoxication rating scale, (2) body temperature assessment, (3) tail blood collection, (4) accelerating rotarod assessment, (5) tilting plane assessment, and (6) loss of righting reflex (LORR) assessment. Across cumulative ethanol doses, adolescent and adult rats evidenced progressive changes in ERB measures. On the ERB, adolescent rats of both sexes evidenced (1) lower intoxication rating, (2) blunted hypothermic responses, particularly in females, (3) longer latencies to fall from the accelerating rotarod, and (4) less tilting plane impairment relative to adults despite comparable BECs. All adult rats, regardless of sex, displayed a LORR at the 3.0 g/kg cumulative ethanol dose while among the adolescent rats, only one male rat and no females showed the LORR. These data reveal decreased adolescent ethanol responsivity across body temperature, intoxication, balance, and coordination responses to a cumulative ethanol challenge as assessed using the novel ERB relative to adults. The results of this study suggest that adolescent-specific low ethanol responsivity may contribute to adolescent binge drinking and increased risk for development of an AUD.

## Introduction

The acute alcohol intoxication response has important medical and legal implications, but few studies to date have linked differences in human acute subjective responses to alcohol as they relate to risk for development of alcohol use disorder (AUD) and the balance, motor, and other tests developed to assess intoxication for enforcement of driving while intoxicated laws. Alcohol tolerance is commonly endorsed as a symptom of AUD [1, 2], but it is unclear how low alcohol responsivity relates to acute responses to alcohol. Low responsivity to alcohol is a strong predictor of heavy drinking and AUD development [3]. There is compelling evidence from multiple groups that low responsivity to alcohol early in life (i.e., adolescence) correlates with later development of heavy drinking, alcohol-related problems, and increased risk for development of an AUD [4–9]. Indeed, low levels of alcohol response at rising and peak blood alcohol levels assessed using subjective, balance, hormonal, electrophysiological, and fMRI responses are associated with increased risk for life-long alcohol problems [10–12]. While acute responsivity to alcohol is generally accepted as fundamentally important and there are extensive human and rodent studies on acute alcohol responses, no studies to date have systematically compared multiple responses to acute cumulative alcohol doses across development [13].

Both human and animal studies support age-related and genetic factors influencing acute alcohol responses. However, most rodent ethanol response assessments only measure a single response. For example, following an acute ethanol challenge, adolescent rats compared to adults display (1) a shorter duration of loss of righting reflex (LORR) and/or elevated blood ethanol levels upon recovery from ethanol-induced sedation [14–18], (2) decreased motor impairment [19–22], (3) differences in social interaction [23], and (4) blunted ethanol-induced hypothermia [24]. The majority of these studies employed a single ethanol dose and assessed one endpoint assuming that alterations in ethanol responses on a given measure are indicative of overall responsivity. However, studies reporting strong genetic contributions to LORR across mouse and rat lines bred to differ markedly in LORR to high-dose ethanol [25–27] suggest that genes influencing LORR sensitivity in rodents do not overlap substantially with those affecting other measures of physical intoxication across inbred strains [28] or in the BXD RI lines [29]. Furthermore, LORR does not appear to be genetically correlated with ethanol drinking or withdrawal in rodents such as the WSP and WSR lines [13, 30]. These studies prompted us to determine how representative of overall acute ethanol responsivity a battery of measures are at a single dose of ethanol across cumulative blood ethanol concentrations.

In an effort to thoroughly evaluate developmental differences in ethanol responsivity across adolescent and adult rats, we developed an ethanol response battery (ERB) to measure acute ethanol responses across cumulative doses of ethanol during the rising phase of the blood ethanol curve. We used the ERB to test the hypothesis that adolescent rats, regardless of sex, evidence low ethanol responsivity relative to adults. The rising phase of blood ethanol is linked to increased reward in brain stimulation threshold in rodents [31, 32]. The ERB assesses intoxication rating, body temperature, balance (rotarod), motor coordination (tilting plane), and onset of LORR endpoints across rising blood ethanol concentrations (i.e., 0.0, 1.0, 2.0, and 3.0 g/kg) in adolescent and adult male and female Wistar rats. The findings reported here replicate and extend previous studies conducted primarily in males with single endpoints further supporting lower ethanol responsivity to acute ethanol intoxication in adolescent female and male rats relative to adults. The ERB may provide a model to better understand and quantitate the mechanisms of acute ethanol responsivity.

## Materials and methods

### Animals

Adolescent (postnatal day [P]30; N = 12 [6 female; 6 male]) and adult (P75; N = 12 [6 female; 6 male]) Wistar rats were obtained from Envigo Laboratories (Indianapolis, IN, USA). Animals were housed in a temperature- (20°C) and humidity-controlled vivarium on a 12/12 h light/dark cycle (light onset at 7:00 AM), and provided *ad libitum* access to food and water. Ethanol-naïve animals were habituated to the vivarium at the University of North Carolina at Chapel Hill for 10 days prior to assessment on the ERB. Ethanol responsivity of female and male adolescent (P40) rats were compared to that of female and male adult (P85) rats using the ERB. Experimental procedures reported in this study were approved by the Institutional Animal Care and Use Committee of the University of North Carolina at Chapel Hill (Protocol #: 20-189). This study was performed in an AAALAC-accredited facility and conducted in strict accordance with NIH regulations for the care and use of animals in research.

### Ethanol response battery

**FIGURE 1:**
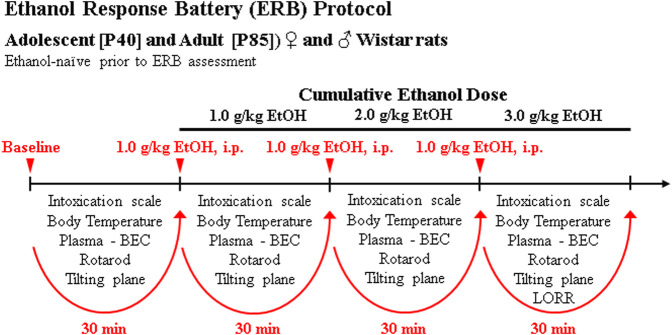
Schematic of ethanol response battery (ERB) protocol. Ethanol-naïve male and female adolescent (P40) and adult (P85) Wistar rats were assessed on the ERB. Each trial of the ERB consisted of (1) the 6-point behavioral intoxication rating scale, (2) body temperature assessment, (3) tail blood collection, (4) accelerating rotarod assessment, (5) tilting plane assessment, and (6) loss of righting reflex (LORR) following the final ethanol dose. The ERB was conducted during a cumulative ethanol dose-response challenge. Following baseline ERB assessment, rats received ethanol doses (1.0, 1.0, 1.0 g/kg, i.p.) approximately 30 min apart for cumulative ethanol doses of 1.0, 2.0, and 3.0 g/kg) with the ERB initiated 15 min following each ethanol dose.

On P40 and P85, animals underwent ERB assessment beginning at 8:00 AM during the light cycle consisting of a baseline assessment followed by three consecutive cumulative doses of ethanol to assess ethanol responsivity across a broad range of blood ethanol concentrations (BECs). The three consecutive doses of ethanol (i.e., 1.0, 1.0, 1.0 g/kg, i.p.) produce cumulative ethanol doses of approximately 1.0, 2.0, and 3.0 g/kg (see Figure 1) that mimic moderate, binge, and heavy drinking blood levels of ethanol. Each subsequent ERB assessment was conducted approximately 30 min apart, each initiated 15 min after ethanol dosing. The ERB consisted of (1) the 6-point behavioral intoxication rating scale, (2) body temperature assessment, (3) tail blood collection, (4) rotarod assessment, (5) tilting plane assessment, and (6) LORR following the final ethanol dose. Body weight was assessed at the beginning of the ERB.

### Behavioral intoxication rating scale

The 6-point behavioral intoxication rating scale was conducted as previously described [33]. Briefly, animals were scored by two researchers according to the following behavioral scale: 1) no sign of intoxication; 2) hypoactivity; 3) slight intoxication (ataxia; slight motor impairment); 4) moderate intoxication (obvious motor impairment; dragging abdomen); 5) high intoxication (dragging abdomen; LORR); 6) extreme intoxication (LORR; loss of eye blink response). The behavioral intoxication rating scale was conducted at baseline as well as 15 min after each ethanol dose for a total of four assessments.

### Body temperature

Body temperature was assessed using a Thermalert clinical monitoring thermometer (Physitemp, Clifton, NJ) with an electric thermometer probe inserted approximately 5 mm into the rectum and left in place for ≥45 s until a stable reading was obtained. Animals were briefly restrained for 2 min in a DecapiCone (ThermoFisher Scientific, Austin, TX) and body temperature was assessed at baseline and again following each ethanol dose following completion of the behavioral intoxication rating scale for a total of four assessments. Difference (Δ) in body temperature as a consequence of cumulative ethanol dosing was calculated by subtracting body temperature following each ethanol dose from baseline body temperature. Room temperature was monitored daily and averaged 20.5°C (range 20°C–21°C).

### Tail blood collection

Tail blood was collected at baseline and 15 min after each administration of ethanol following body temperature assessment to determine BECs using a GM7 Analyzer (Analox; London, United Kingdom).

### Accelerating rotarod

Adolescent and adult rats were trained on the accelerating rotarod (IITC Life Sciences, Woodland Hills, CA) for 2 days (three trials per day) prior to ERB assessment. The rotarod cylinder was 9.5 cm in diameter and 15 cm wide. On Training Day 1, each animal received three 3 min training trials with Trial 1 at 5 rotations per minute (rpm), Trial 2 at 10 rpm, and Trial 3 consisting of a start speed of 5 rpm and accelerating to 20 rpm over 3 min. If the animal fell off the rotarod cylinder during Training Day 1, the animal was gently placed back onto the cylinder for the remainder of training. Twenty-four h later on Training Day 2, each animal received an additional three 3 min training trials with Trial 1 at 10 rpm followed by two consecutive trials with the rotarod cylinder with a start speed of 5 rpm and accelerating to 20 rpm over 3 min. By the conclusion of rotarod training, nearly all adolescent and adult male (time on rotarod: adolescent: 177 s, adult: 178 s; *t*[10] = 0.44, *p* = 0.67) and female (time on rotarod: adolescent: 157 s, adult: 157 s; *t*[10] = 0.00, *p* = 0.99) rats were able to remain on the rotarod cylinder for the entirety of the final 3 min training session.

Twenty-four h later, rotarod performance was assessed during the ERB and each session consisted of three successive trials on the accelerating rotarod with a start speed of 5 rpm and accelerating to 20 rpm over 3 min. Latency to fall was recorded for every trial and was the primary outcome measure of the rotarod. Time (s) spent on the rotarod was assessed at baseline and again following each ethanol dose after completion of tail blood collection for a total of four sessions. Each of the three successive trials per dosing session were averaged and change from baseline (Δ) in time spent on the rotarod as a consequence of cumulative ethanol dosing was calculated by subtracting time on the rotarod during baseline performance from each ethanol dose rotarod performance.

### Tilting plane

The tilting plane apparatus consisted of a clear Plexiglas box (60 cm × 24 cm × 20 cm) attached with a hinge to a frame with a glass panel floor. The box was tilted via an additional hinge attached to the base and a sliding protractor was used to measure the angle at which subjects began to slide down the glass floor panel. At the time of testing, the rat was placed on the apparatus facing away from the tilting hinge and the panel lifted slowly until the subject began to slide down the floor of the apparatus. The angle at which the rat began to slide was measured and the procedure repeated for three consecutive trials per session. The three trials within each session were averaged and difference (Δ) in angle of slide as a consequence of cumulative ethanol dosing was calculated by subtracting angle of slide from the averaged baseline angle of slide from each ethanol dose angle of slide.

### Loss of righting reflex

Loss of righting reflex was assessed following the final dose of ethanol after completion of the tilting plane. Animals were placed on their back in a V-shaped trough and assessed for righting reflex. Loss of righting reflex was defined as the inability of the rat to right itself onto all four paws within 60 s.

### Statistical analysis

Statistical analysis was performed using GraphPad Prism 8 (San Diego, CA). Body weight and baseline measures were assessed using two-tailed Student’s *t* tests. Levene’s test for equality of variance was performed for each analysis. When reported in the Results, Welch’s *t* tests were used to assess data with unequal variances. BECs, intoxication rating, body temperature, rotarod, and tilting plane data were first assessed using the Shapiro-Wilk test to determine normality of data distribution. Data with a normal distribution were then assessed using parametric 2 × 2 repeated measure ANOVAs with post-hoc Šidák’s multiple comparison tests performed when appropriate. Data that was not normally distributed was assessed using the non-parametric Mann-Whitney test. Ordinal data (i.e., intoxication rating) was assessed using the Mann-Whitney test. Pearson Chi Square was used to analyze LORR data. All values except intoxication rating are reported as mean ±SEM. The intoxication rating data is reported as median with the interquartile range.

## Results

**FIGURE 2:**
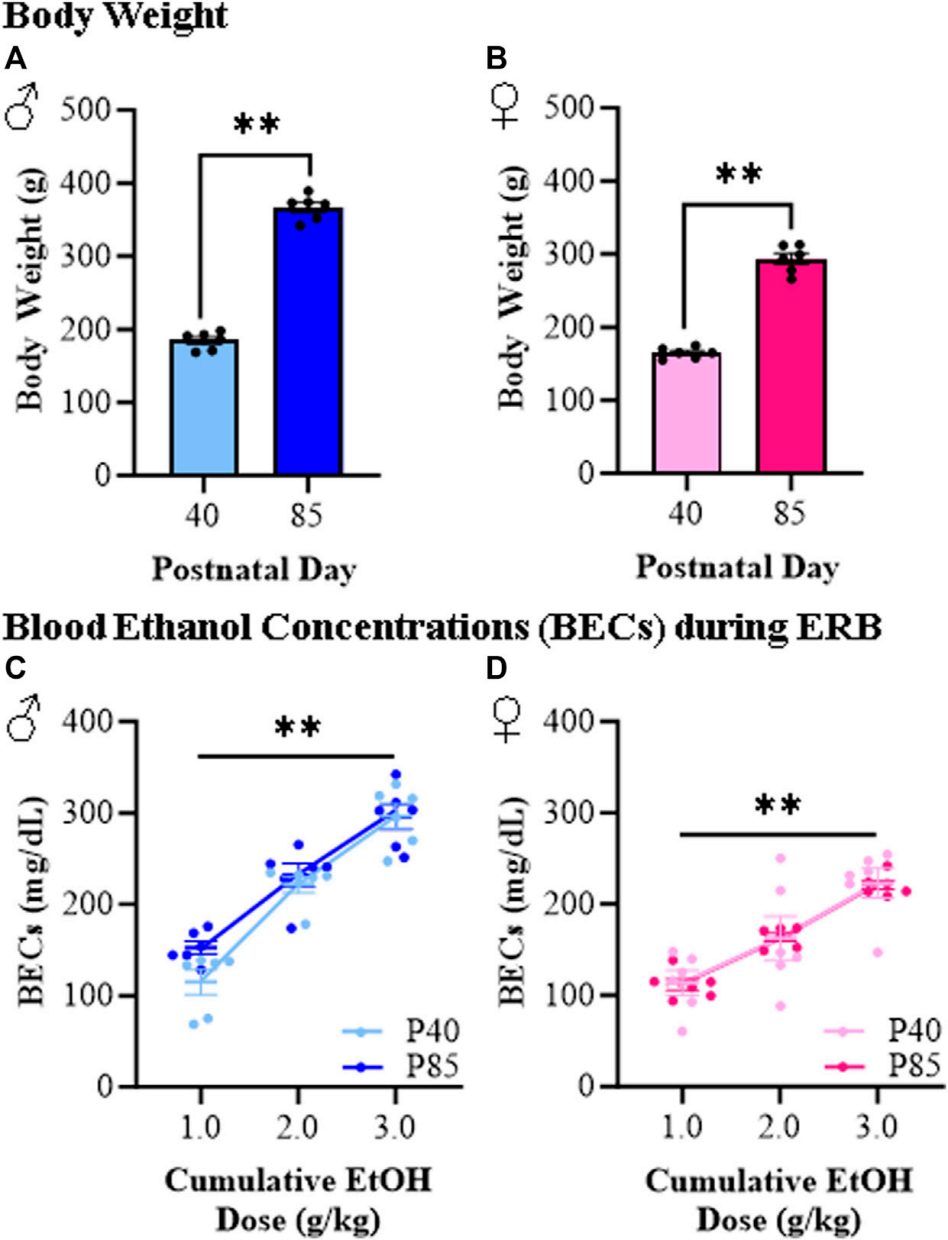
Body weight and blood ethanol concentration (BEC) assessment during the ethanol response battery (ERB) across the cumulative ethanol challenge. **(A)** Adolescent (P40) male subjects weighed significantly less than adult (P85) male subjects (*t*[10] = 21.5, *p* < 0.0001, Student’s t*-*test). **(B)** Adolescent (P40) female subjects weighed significantly less than adult (P85) female subjects (*t*[10] = 15.7, *p* < 0.0001, Student’s t*-*test). **(C)** Assessment of BECs 15 min after each ethanol dose across the cumulative ethanol challenge during ERB testing in male subjects reveal a dose-dependent increase in BECs (main effect of Dose: *F*(_2,20_) = 102.3, *p* < 0.0001, repeated measures ANOVA) that did not differ as a function of age (main effect of Age: *p* = 0.15, repeated measures ANOVA). **(D)** Assessment of BECs in female subjects 15 min after each ethanol dose across the cumulative ethanol challenge revealed a dose-dependent increase in BECs (main effect of Dose: *F*(_2,20_) = 85.0, *p* < 0.0001, repeated measures ANOVA) that was unaffected by age (main effect of Age: *p* = 0.96, repeated measures ANOVA). *n* = 6 subjects/age/sex. Data are presented as mean ±SEM. **p < 0.01.

To better understand how responses to ethanol differ between adolescent and adult female and male rats, we developed an ERB to assess ethanol responsivity across cumulative ethanol doses (i.e., 0.0, 1.0, 2.0, and 3.0 g/kg) allowing quantitative determination of developmental dose response curves. As expected, male rats were bigger than females, and adolescent (P40) rats of both sexes weighed significantly less than adult (P85) rats (see Figures 2A, B). Assessment of blood ethanol levels during ERB cumulative ethanol doses revealed remarkably similar progressive increases in BECs across age groups from approximately 100 mg/dL to approximately 250–300 mg/dL in both adolescent and adult rats (see Figures 2C, D). The observed similarities in BECs across adolescent and adult rats provided a strong rationale for comparison of ERB performance across ages. Unfortunately, BECs differed significantly between the male and female rats, regardless of age, preventing direct comparisons of sex differences.

**FIGURE 3:**
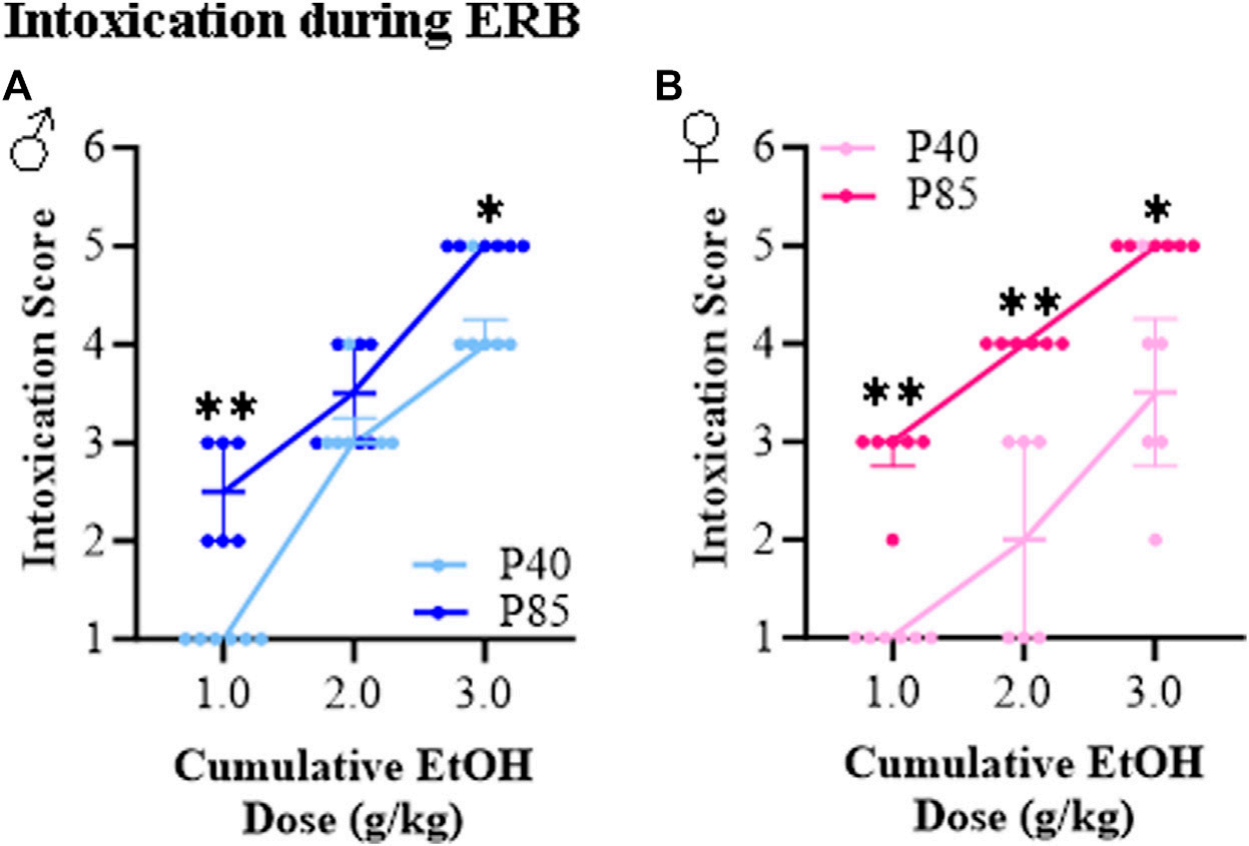
Ethanol response battery (ERB) assessment revealed an adolescent-associated reduction in intoxication rating to a cumulative ethanol challenge relative to adults. **(A)** Across adolescent (P40) and adult (P85) male subjects, assessment of intoxication revealed that adolescents had lower intoxication rating scores at the 1.0 g/kg (*U* = 0.00, *p* = 0.002, Mann-Whitney test) and 3.0 g/kg (*U* = 3.00, *p* = 0.015, Mann-Whitney test) dose, but not at the 2.0 g/kg (*U* = 12.00, *p* = 0.394, Mann-Whitney test) dose relative to male adults. **(B)** Across adolescent (P40) and adult (P85) female subjects, assessment of intoxication revealed that adolescents had lower intoxication rating scores at the 1.0 g/kg (*U* = 0.00, *p* = 0.002, Mann-Whitney test), 2.0 g/kg (*U* = 0.00, *p* = 0.002, Mann-Whitney test), and 3.0 g/kg (*U* = 3.00, *p* = 0.015, Mann-Whitney test) doses relative to female adults. *n* = 6 subjects/age/sex. Data are presented as median with interquartile range. *p < 0.05, **p < 0.01.

The ethanol intoxication rating scale has been used in binge drinking models for many years to provide a measure of intoxication [33, 34]. At the acute ethanol dose of 1.0 g/kg, all groups achieved BECs of approximately 100–130 mg/dL, yet adolescent (P40) male and female rats all exhibited a normal appearance as indicated by an intoxication rating of 1.0 (±0.0). This contrasts with adult (P85) rats, which despite having BECs similar to adolescents, showed intoxication scores of 2.5 (±0.2) in males and 2.9 (±0.2) in females consistent with hypoactivity and slight intoxication. Across cumulative ethanol doses, both adolescent and adult rats evidenced increasing intoxication scores with rising BECs (see Figures 3A, B). However, the dose response curve of adolescents differed significantly from that of adults, with adolescents of both sexes demonstrating lower intoxication scores than adults consistent with adolescents showing less intoxication at comparable BECs. Both male and female adult rats evidenced intoxication scores of 5.0 (±0.0) at the highest alcohol dose (i.e., 3.0 g/kg) indicating high intoxication as evidenced by dragging of the abdomen and/or LORR, but not loss of eye blink reflex. In contrast, intoxication rating scores of 4.3 (±0.2) and 3.7 (±0.4) were observed at the 3.0 g/kg dose in the adolescent male and female rats, respectively. Thus, both adolescent male and female rats show lower intoxication scores across a broad range of ethanol doses relative to adults.

**FIGURE 4:**
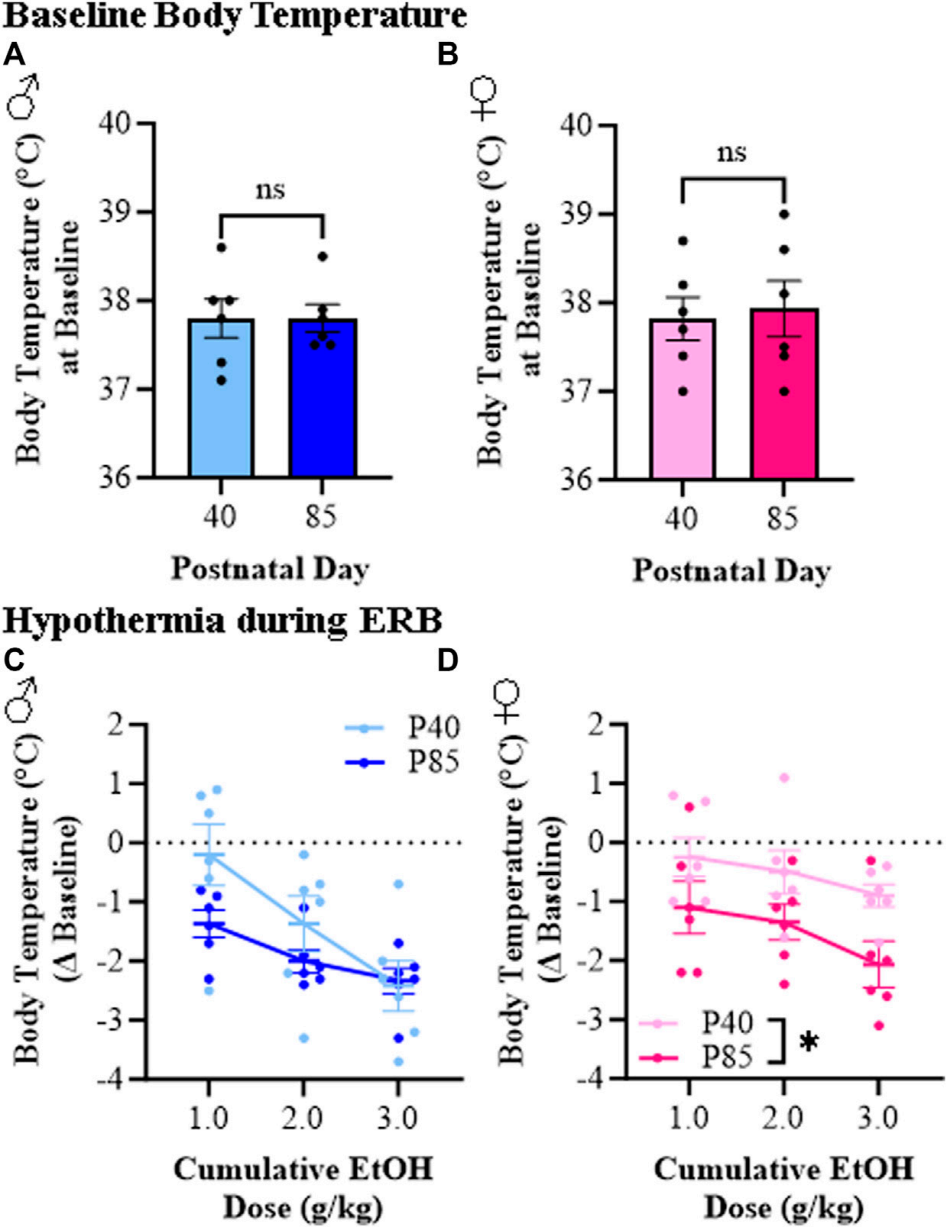
Ethanol response battery (ERB) assessment revealed a female-specific adolescent-associated reduction in hypothermic responsivity to a cumulative ethanol challenge relative to adults. **(A)** Body temperature assessment at baseline did not differ between adolescent and adult male subjects (*t*[10] = 0.0, *p* = 0.99, Student’s t*-*test). **(B)** Body temperature assessment at baseline did not differ between adolescent and adult female subjects (*t*[10] = 0.29, *p* = 0.78, Student’s t*-*test). **(C)** Assessment of body temperature following cumulative ethanol challenge dosing in male subjects revealed a dose-dependent hypothermic response across ethanol doses (main effect of Dose: *F*(_2,20_) = 12.7, *p* = 0.003, repeated measures ANOVA) that was insignificantly blunted in adolescent male rats relative to adult males (main effect of Age: *F*(_1,20_) = 2.4, *p* = 0.15, repeated measures ANOVA). **(D)** Assessment of body temperature following cumulative ethanol challenge dosing in female subjects revealed a dose-dependent hypothermic response across cumulative ethanol doses (main effect of Dose: *F*(_2,20_) = 10.4, *p* = 0.001, repeated measures ANOVA) as well as a tread toward reduced ethanol-induced hypothermia in adolescent female subject rats relative to adult females (main effect of Age: *F*(_1,20_) = 4.6, *p* = 0.056, repeated measures ANOVA). *n* = 6 subjects/age/sex. Data are presented as mean ±SEM.

Hypothermia is a commonly studied ethanol response endpoint [35]. While body temperature did not differ at baseline as a function of sex or age (see Figures 4A, B), we observed differences in ethanol-induced hypothermia between adolescent (P40) and adult (P85) rats (see Figures 4C, D). At the 1.0 g/kg dose, adolescent rats evidenced minor reductions in body temperature (male: −0.2°C [±0.5°C]; female: −0.3°C [±0.3°C] from baseline) whereas adults of both sexes demonstrated an approximate 1.0°C drop in body temperature (male: −1.4°C [±0.2°C]; female: −1.1°C [±0.4°C] from baseline). The cumulative ethanol dosing paradigm resulted in a dose-dependent reduction in body temperature with the highest dose causing an approximate 2.0°C–3.0°C drop across all groups except adolescent females, who evidenced a maximum drop of approximately 1.0°C. Thus, the hypothermic response to ethanol is dose-dependent in both male and female adolescent and adult rats whereas adolescents overall show a reduced hypothermic response, particularly in adolescent females, to a cumulative ethanol dosing challenge.

**FIGURE 5:**
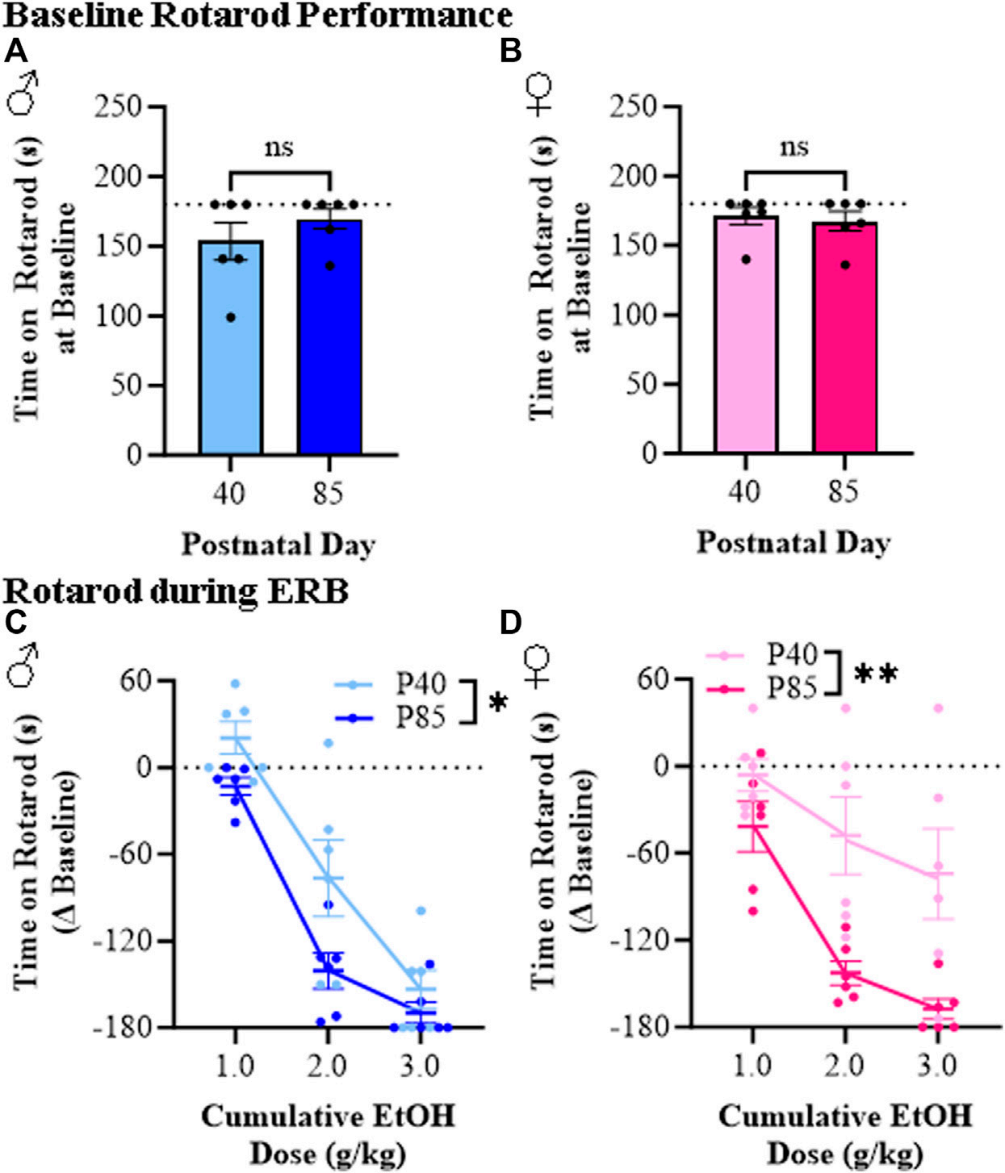
Ethanol response battery assessment revealed an adolescent-associated reduced sensitivity to impairment on the rotarod across the cumulative ethanol challenge relative to adults. **(A)** Baseline performance on the rotarod did not differ between adolescent and adult male subjects (*t*[10] = 1.1, *p* = 0.32, Student’s t*-*test). **(B)** Baseline performance on the rotarod did not differ between adolescent and adult female subjects (*t*[10] = 0.38, *p* = 0.71, Student’s t*-*test). **(C)** Across adolescent (P40) and adult (P85) male subjects, assessment of rotarod performance revealed a dose-dependent impairment in time to remain on the rotarod across cumulative ethanol doses (main effect of Dose: *F*(_2,20_) = 162.5, *p* < 0.0001, repeated measures ANOVA). Relative to adult male rats, adolescent males spent significantly more time on the rotarod across the cumulative ethanol challenge (main effect of Age: *F*(_1,20_) = 4.8, *p* = 0.052, repeated measures ANOVA). **(D)** Across adolescent and adult female subjects, time on the rotarod was reduced across cumulative ethanol doses (main effect of Dose: *F*(_2,20_) = 21.6, *p* < 0.0001, repeated measures ANOVA). Adolescent female rats, regardless of ethanol dose, were less impaired on the rotarod relative to adult females (main effect of Age: *F*(_1,20_) = 13.0, *p* = 0.005, repeated measures ANOVA). *n* = 6 subjects/age/sex. Data are presented as mean ±SEM. *p ≤ 0.05, **p < 0.01.

Rotarod performance was assessed during ERB testing to measure balance between adolescent and adult male and female rats across cumulative ethanol doses. While time on the accelerating rotarod did not differ at baseline across sex or age (see Figures 5A, B), latency to fall across all groups relative to baseline progressively decreased as the ethanol dose increased (see Figures 5C, D). Interestingly, both male and female adolescent rats spent consistently more time on the accelerating rotarod (i.e., demonstrated better balance) than adult males and females. As an example, at the 2.0 g/kg dose of ethanol, adolescent male and female rats remained on the rotarod for approximately 77 s (±26 s) and 123 s (±27 s), respectively, whereas time on the rotarod in adult males (29 s [±12]) and adult females (25 [±8]) was significantly less despite comparable BECs. Female adolescent rats in particular were markedly less impaired at the 3.0 g/kg ethanol dose than adult female rats. Thus, these rotarod studies provide further support for a reduced sensitivity to the effect of ethanol on balance for adolescent (P40) compared to adult (P85) male and female rats.

**FIGURE 6:**
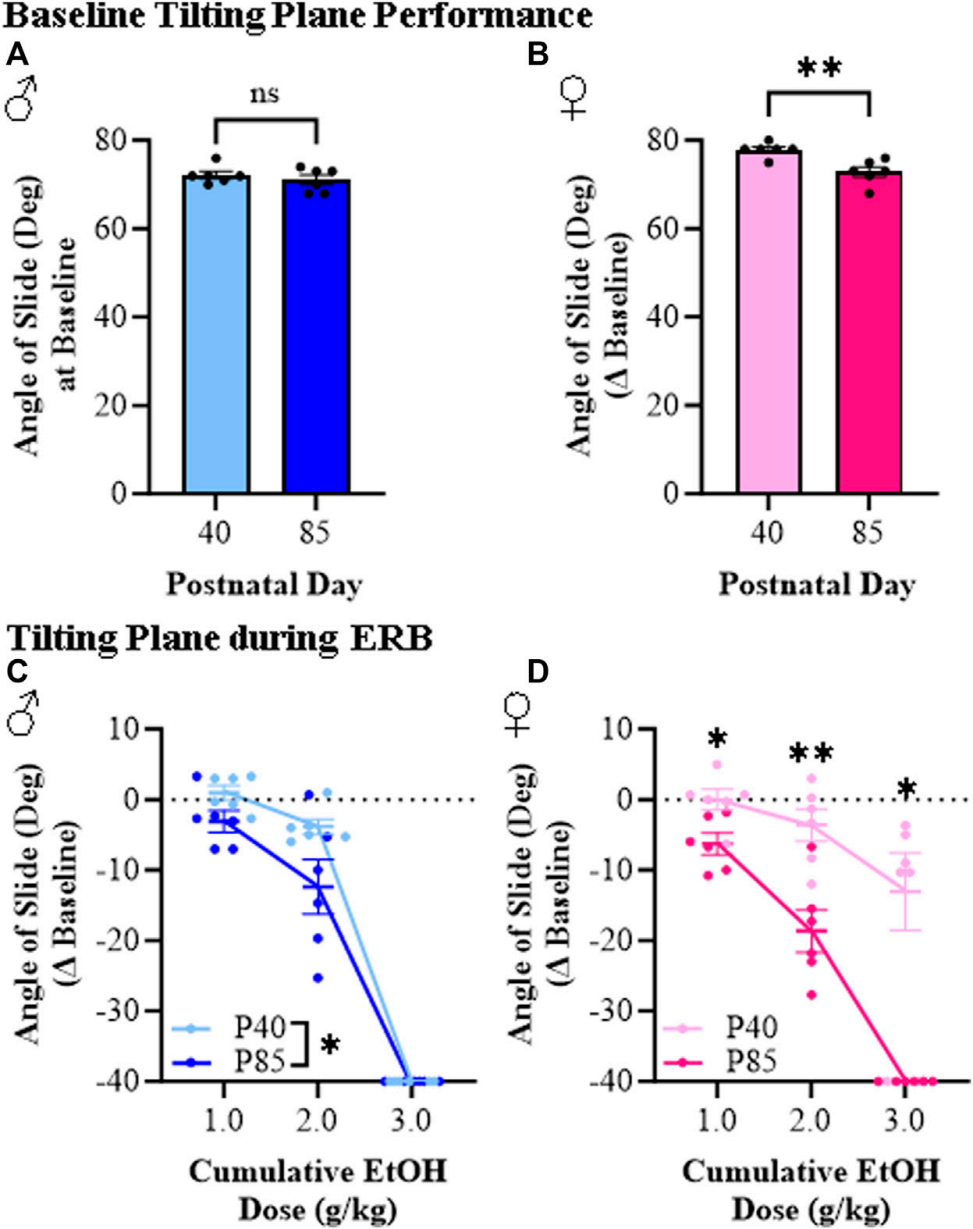
Ethanol response battery assessment revealed an adolescent-associated reduced sensitivity to impairment on the tilting plane across the cumulative ethanol challenge relative to adults. **(A)** Baseline performance on the tilting plane did not differ between adolescent and adult male subjects (*t*[10] = 0.85, *p* = 0.42, Student’s t*-*test). **(B)** At baseline, adolescent female rats performed slightly better on the tilting plane than adult females (*t*[10] = 3.8, *p* = 0.003, Student’s t*-*test). **(C)** Assessment of tilting plane performance in male subjects revealed a dose-dependent reduction in angle of slide across cumulative ethanol doses (main effect of Dose: *F*(_2,20_) = 344.4, *p* < 0.0001, repeated measures ANOVA) whereas adolescents, regardless of ethanol dose, were less impaired on the tilting plane than adults (main effect of Age: *F*(_1,20_) = 5.5, *p* = 0.041, repeated measures ANOVA). **(D)** Assessment of tilting plane performance in female subjects revealed a dose-dependent reduction in angle of slide across cumulative ethanol doses (main effect of Dose: *F*(_2,20_) = 40.1, *p* < 0.0001, repeated measures ANOVA) whereas adolescents were overall less impaired on the tilting plane relative to adults (main effect of Age: *F*(_1,20_) = 37.4, *p* = 0.009, repeated measures ANOVA). Follow-up posthoc analysis of the significant Dose × Age interaction (*F*(_2,20_) = 7.67, *p* = 0.003, repeated measures ANOVA) revealed less impairment in adolescent female rats relative to adult females at cumulative ethanol doses of 1.0 g/kg (*p* = 0.045, Šidák’s multiple comparisons test), 2.0 g/kg (*p* = 0.008, Šidák’s multiple comparisons test), and 3.0 g/kg (*p* = 0.013, Šidák’s multiple comparisons test). Data are presented as mean ±SEM. *p ≤ 0.05, **p < 0.01.

The tilting plane is often used to assess motor coordination, which provides a measure of the ability of rodents to maintain balance as the angle of the horizontal plane is gradually increased. Baseline performance on the tilting plane did not differ between adolescent and adult male subjects (see Figure 6A) whereas adolescent female rats performed slightly better than adults at baseline (see Figure 6B). Across the cumulative ethanol dosing, we observed a dose-dependent reduction in the angle of slide across adolescent and adult male and female rats (see Figures 6C, D). Interestingly, both adolescent male and female rats were less impaired than adults of both sexes at the 1.0 g/kg and 2.0 g/kg doses of ethanol despite comparable BECs across ages. For example, at the 2.0 g/kg dose, angle of slide decreased 3.8° (±1.0°) and 3.6° (±2.3°) in adolescent male and female rats, respectively, whereas the angle of slide decreased 12.4° (±3.9°) in adult males and 18.7° (±3.0°) in adult females despite comparable BECs. However, at the highest dose (i.e., 3.0 g/kg), adolescent and adult male rats were equally impaired on the tilting plane while adolescent females continued to evidence reduced sensitivity relative to adult females. These findings are consistent with reduced ethanol responsivity in adolescent rats compared to adult rats across sexes.

**FIGURE 7:**
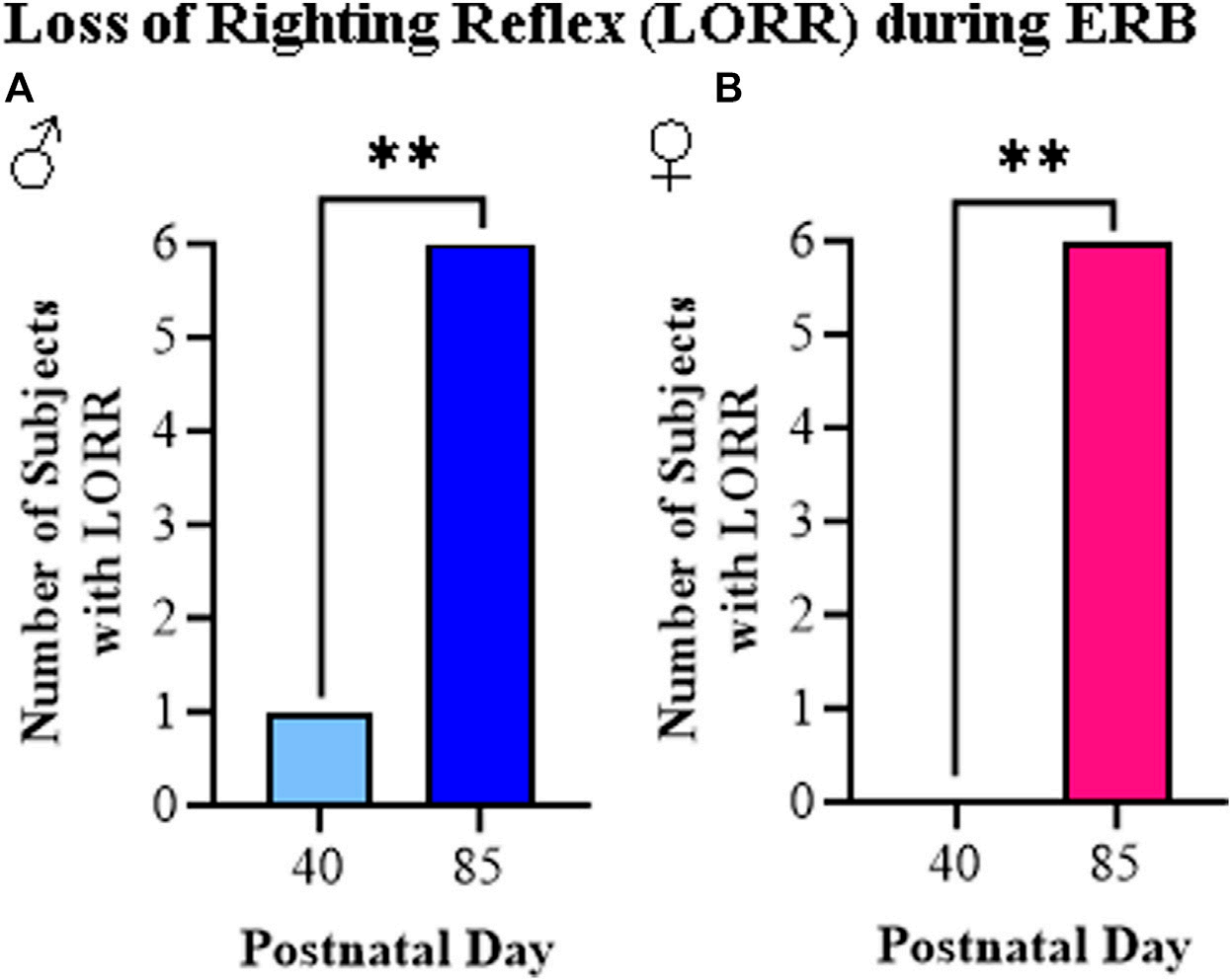
The ethanol response battery revealed an adolescent-associated insensitivity to loss of righting reflex (LORR) relative to adults. **(A)** Assessment of LORR in male rats following the final cumulative dose of ethanol (i.e., 3.0 g/kg) revealed that only one adolescent displayed LORR whereas all of the adults evidenced LORR (*X* ^*2*^(1, *N* = 12) = 8.6, *p* = 0.003, Pearson Chi Square). **(B)** Assessment of LORR in female rats following the final cumulative dose of ethanol (i.e., 3.0 g/kg) revealed that no adolescents displayed LORR whereas all of the adults evidenced LORR (*X* ^*2*^(1, *N* = 12) = 12, *p* = 0.0005, Pearson Chi Square). *n* = 6 subjects/age/sex. Data are presented as number of subjects with LORR. **p < 0.01.

The loss of righting reflex is a measure of the sedative actions of ethanol as determined by the ability of a rat placed on its back to turn and upright itself. In our study, we assessed LORR just after completing the final dose of ethanol (i.e., 3.0 g/kg). Consistent with prior studies, all adult rats, regardless of sex, displayed a LORR while among the adolescent rats, only one male rat (see Figure 7A) and no female rats (see Figure 7B) showed the LORR. Thus, the LORR assessment demonstrates a robust difference between adolescent and adult rat responsivity to ethanol regardless of sex.

## Discussion

In the present study, we designed an ERB to assess alcohol responsivity across a broad range of cumulative ethanol doses (i.e., 0.0, 1.0, 2.0, and 3.0 g/kg) to provide a quantitative determination of developmental dose response curves in adolescent (P40) male and female Wistar rats relative to adults (P85). Through cumulative ethanol doses spaced 30 min apart, we report progressive increases in BECs from approximately 100 mg/dL to 250–300 mg/dL that were remarkably similar across adolescent and adult male and female rats. The ERB determinations of intoxication rating, body temperature, accelerating rotarod, and tilting plane all demonstrated progressive changes with increasing BECs. Although the intoxication rating and hypothermia measures show increased sensitivity at lower ethanol doses than the accelerating rotarod and tilting plane, all of the ERB measures show a progressive ethanol dose response curve suggesting a common mechanism. While a BEC at 100 mg/dL is above the human legal driving limit, we did not observe impairment on any of these measures in adolescent male and female rats relative to adults. Previous studies using chronic ethanol vapor administration during adolescence found adolescent male rats with an approximate 200 mg/dL BEC demonstrated an intoxication rating of 1 (i.e., no signs of intoxication) [36]. Here, we replicate and extend these studies to include females as well as measures of hypothermia, accelerating rotarod, and tilting plane across a cumulative ethanol dosing challenge, all of which show no response to the lowest dose of ethanol (i.e., 1.0 g/kg) in adolescent males and females. Further, male and female adults rats (P85) do not show accelerating rotarod or tilting plane responses at the lowest ethanol dose. In contrast, at the highest cumulative dose of ethanol (i.e., 3.0 g/kg), adult male and females are highly intoxicated, rapidly falling off of the rotarod, unable to maintain balance on the tilting plane, and demonstrating LORR. Thus, the ERB provides acute ethanol dose response curves across a spectrum of BECs from little to no effect to dramatic intoxication. The comparable BECs observed at the time of ERB testing across ages suggest that the observed lower ethanol responsivity in adolescent rats was not attributable to an increase in ethanol metabolism. Our findings include both sexes and are consistent with many previous studies administering a single ethanol dose and assessing one response endpoint to compare ethanol responsivity across adolescent and adult male rats.

Studies in both humans and rodents have generally reported that alcohol decreases body temperature [37–39]. We found ethanol induced a reduction in body temperature (i.e., hypothermia) to a greater degree in adult rats than was observed in adolescent rats. Although studies generally agree that acute ethanol administration induces hypothermia, the outcomes of these studies are inconsistent and vary depending on multiple factors including the age of adult animals assessed as well as the timing of assessment following ethanol administration. Indeed, acute ethanol administration was previously reported to cause similar reductions in core body temperature in adolescent and adult rats [21, 24], increase hypothermia in adolescent rats compared to adults [40–42], increase hypothermia in aged rats compared to adult rats [43], and decrease hypothermia in adolescent rats compared to adults [35]. These conflicting results appear to be due to different doses being administered (lower ethanol dose vs. higher ethanol dose), the researchers’ handling of the animals during experimentation, the timing of assessment, and the ambient room temperature [38]. For example, a recent study of ethanol-induced hypothermia reported similar results to our studies of increased sensitivity to ethanol with increasing age, comparing adolescent, adult, and aged rats [39]. While similar doses of ethanol were employed in that study (i.e., 1.0, 2.0, 3.0 g/kg), the study timing of dosing and measures differed. The design was to separate each ethanol dose across a 21 day period in a mixed design, with BECs and hypothermia assessed from 60–360 min following each individual dose. In contrast, our ERB is designed to assess acute ethanol across cumulative ethanol doses every 30 min during the rising blood ethanol levels to mimic an acute binge drinking episode and assess responsivity across multiple doses in a cumulative fashion, with each assessment occurring within 30 min. While our ERB assessment does not show adolescent differences in BECs just after dosing relative to adults, elimination of ethanol over several hours increases BEC variability over time and response changes over time during exposure to ethanol that increase variability within and across experimental groups. Our cumulative ethanol dosing regimen and focus on acute responses during rising blood ethanol levels benefits from remarkably consistent BECs across groups of varying age, sex, and body weights. Thus, our findings of a reduced hypothermic response to a cumulative ethanol challenge in adolescent male and female rats relative to adults replicate and extend prior studies.

The cumulative ethanol dose response in adult rats results in a progressive increase in intoxication from a normal appearance to LORR responses. The lowest dose of ethanol studied (i.e., 1.0 g/kg) showed the largest differences in intoxication rating and hypothermia between adolescent (P40) and adult (P85) rats although, in general, the entire dose response for both male and female adolescent rats differed from adults. In contrast, the rotarod and tilting plane showed the largest differences across age following the 2.0 g/kg ethanol dose. Differences in responsivity across the cumulative ethanol challenge for different measures are not surprising since it would be expected that hypothermic response might reflect a hypothalamic response whereas balance and motor coordination might involve different brain regions. For example, a recent study in adult mice found ethanol-induced rotarod impairments related to cerebellar ethanol metabolism-induced elevation of GABA levels [44]. Thus, different brain regions and mechanisms may reflect the differences across ethanol dose responsivity measures. Rotarod responses in females were particularly blunted at higher BECs, with adolescent female rats remaining on the rotarod and not showing a LORR. Females, both adolescent and adult, have a slightly lower maximal BEC than males, likely contributing to the difference in adolescent male and female rotarod and tilting plane performance at the higher BECs. Although these motor responses at high ethanol doses show distinct adolescent and adult differences, a weakness of the ERB is that the lowest ethanol dose administered achieves BECs consistent with binge drinking levels of ethanol. Human studies of alcohol responsivity generally produce blood alcohol levels at binge drinking levels and have reported subjective and motor differences in acute ethanol responses, although subjective responses show the most robust association with progressive increases in drinking and development of AUD [45]. While one might conclude, based on the preclinical and clinical studies, that rats are less sensitive to ethanol in general, studies of brain reward stimulation in rodents report ethanol at levels below 100 mg/dL can enhance reward [31, 32]. Thus, in future studies we plan to include lower ethanol doses to explore responsivity across a broader range of blood ethanol levels to provide a better translational assessment of acute ethanol responses.

Many studies have previously examined the ethanol-induced LORR, which is an anesthetic-like response to a high dose of ethanol and involves the ability of a rodent to right itself onto all four paws after placement on its back. The duration of the LORR (or, more accurately, the alcohol concentration at which the righting reflex is lost or regained) differs substantially across genotypes [26, 27, 29]. Human and rodent low responsivity to alcohol are important as a low alcohol response in humans is linked to increased risk of AUD development. The LORR after an anesthetic dose of alcohol is quite different from human body sway low responses commonly assessed at relatively low doses of alcohol. While there is a strong genetic contribution to LORR across mouse and rat lines bred to differ markedly in LORR to high-dose ethanol [25, 27], evidence suggests that genes influencing LORR sensitivity in rodents do not overlap substantially with those affecting other measures of physical intoxication across inbred strains [28, 29]. Further, LORR does not appear to be genetically correlated with ethanol drinking or withdrawal in rodent lines such as the WSP or WSR lines [30]. Although our findings of reduced adolescent sensitivity to LORR compared to adults might be particularly important in relation to extreme binge drinking levels engaged in by some adolescents, this could be limited in adults and aged adults due to increases in anesthetic-like sensitivity to high dose ethanol. The mechanisms of increased sensitivity of acute ethanol responses with age are poorly understood.

Adolescence is a developmental period during which both humans and animals are reported to display lower responsivity to many of the aversive effects of alcohol relative to adult counterparts that may convey increased risk for excessive alcohol intake and later development of AUD [46–51]. Clinically, it appears that adolescent individuals are less affected by alcohol withdrawal than adults [52]. Reports in the preclinical literature are much more abundant and specific to these developmental differences. For instance, there is evidence that during early and/or late alcohol withdrawal, adolescent rats relative to adults display (1) lower anxiety-like behavior as assessed using the elevated plus maze [53], (2) attenuated suppression of social interactions ([54], but see Wills et al. [55, 56]), (3) less distress as assessed by measuring ultrasonic vocalizations [40], and (4) decreased seizure intensity (but see [56, 57]). Similarly, following an acute alcohol challenge, adolescent rats relative to adults display (1) a shorter duration of LORR and/or elevated BECs at recovery from alcohol-induced sedation [14, 15, 17, 21] and (2) decreased motor impairment [19–21]. Thus, the findings of the present study replicate and extend previous research demonstrating decreased responsivity across a battery of physiological and behavioral measures to a cumulative ethanol challenge in adolescent male and female rats relative to adults.

In summary, we report decreased ethanol responsivity to a cumulative ethanol challenge as assessed using the novel ERB in adolescent male and female Wistar rats relative to adults. Across cumulative ethanol doses, both adolescent and adult rats evidenced increasing intoxication rating scores with rising BECs whereas adolescents of both sexes demonstrated lower intoxication scores than adults despite comparable BECs. We observed a dose-dependent ethanol-induced hypothermic response in both male and female adolescent and adult rats, but adolescents overall showed a reduced hypothermic response, particularly in adolescent females, to a cumulative ethanol dosing challenge. On the accelerating rotarod, both male and female adolescent rats spent consistently more time (i.e., had better balance) on the rotarod than adult males and females across the cumulative ethanol challenge. Cumulative ethanol dosing led to an expected reduction in the angle of slide across adolescent and adult male and female rats, but adolescent male and female rats were less impaired than adults of both sexes at the 1.0 g/kg and 2.0 g/kg doses of ethanol despite comparable BECs across ages. All adult rats, regardless of sex, displayed a LORR while among the adolescent rats, only one male rat and no females showed the LORR. The findings reported here replicate and extend previous studies conducted primarily in males with single endpoints, further supporting lower ethanol responsivity to acute ethanol intoxication in adolescent female and male rats relative to adults.

## Data Availability

The raw data supporting the conclusion of this article will be made available by the authors, without undue reservation.
